# The Fragile Balance: Autophagy's Role in Neurodegenerative Disease Progression

**DOI:** 10.2174/011570159X377552250627113915

**Published:** 2025-07-03

**Authors:** Bharat Bhushan, Meenakshi Dhanawat, Garima, Kashish Wilson, Sumeet Gupta, Samrat Chauhan

**Affiliations:** 1 Institute of Pharmaceutical Research GLA University, Mathura, 281406, India;; 2 Amity Institute of Pharmacy, Amity University, Amity Education Valley, Panchgaon, Manesar, Gurugram, Haryana, 122413, India;; 3 Chitkara College of Pharmacy, Chitkara University, Rajpura, Punjab, 140401, India;; 4 M.M College of Pharmacy, Maharishi Markandeshwar (Deemed to be University), Mullana-Ambala, Haryana, 133207, India

**Keywords:** Autophagy, cancer, heart disease, liver disease, protein aggregates, autophagy-related gene, neurodegenerative disease

## Abstract

Autophagy relates to the mechanism underlying the intracellular constituents’ breakdown by lysosomes. Autophagy plays an essential role in preserving and regulating cellular homeostasis by mediating the degradation of intracellular components and recycling their decomposition products. It was demonstrated that autophagy operates *in-vivo* in the starving reaction, initial growth, internal control of quality, and cell division. Autophagy malfunction is perhaps connected with cancer and neurological conditions, as demonstrated by current research. In conjunction with the identification of specific mutations associated with autophagy-related disorders and deeper knowledge of the pathophysiology of disorders caused by aberrant disintegration of particular autophagy substrates, autophagy activation serves a vital part in prolonging lifespans and suppressing the process of aging. To safeguard the homeostasis within a cell, cells have developed sophisticated quality-control procedures for organelles and proteins. These quality-control mechanisms maintain cellular integrity through degradation by the autophagy-lysosome or ubiquitin-proteasome systems, as well as through protein folding assistance (or refolding of misfolded proteins) provided by molecular chaperones. A great deal of neurodegenerative illnesses are indicated by the development of intracellular inclusions formed from misfolded proteins, which are believed to be an outcome of defective autophagy. Additionally, it was recently discovered that neurodegenerative illnesses are also linked with mutations in key autophagy-related genes. However, pathogenic proteins like α-synuclein and amyloid β cause damage to the autophagy system. This paper examines the recent advancements in our understanding of the link between autophagic abnormalities and the development of neurological disorders, and proposes that activating autophagy could serve as a potential therapeutic strategy.

## 1 INTRODUCTION

Autophagy is the physiological process through which cells transport their components *via* autophagosomes into Lysosomes for degradation. Christian de Duve discovered several hydrolases in the 1950s as electron microscopy advanced, which helped scientists identify lysosomes as cellular organelles [1]. In 1963, De Duve coined the term “autophagy” to explain how cells combine lysosomes with vesicles containing proteins, directing cellular protein disintegration. We called the vesicles autophagosomes. Autophagy is a term frequently utilized to refer to macroautophagy. The cargo that is destroyed by macroautophagy is capable of being reprocessed into fresh macromolecules.

Autophagosomes are transitory structures with multiple membranes that unite to form vacuoles as well as lysosomes. They encapsulate precursors of cytosolic macro-autophagy. The reuse and recovery of substrates through macro-autophagy is vital to sustaining cellular homeostasis [2]. Chaperone-Mediated Autophagy is distinctive amongst autophagies because it specifically targets proteins during degradation in lysosomes. It was previously believed that the main purpose of this pathway in the metabolism of cells was primarily to transport free amino acids produced throughout the breakdown of proteins. Nevertheless, current research indicates that impaired Chaperone-Mediated Autophagy significantly affects the body's general energy metabolism, insulin, and lipid utilization and regulates cellular metabolism when exposed to different nutrients [3]. In microautophagy, a non-selective lysosomal degradation process, the lysosomal membrane directly invaginates or engulfs portions of the cytoplasm containing cargo, thereby facilitating its degradation within the lysosomal lumen [4]. Moreover, autophagy systems collaborate to generate degradation products and destroy intracellular elements to maintain homeostasis *in-vivo*. Frequent disruption of autophagy can result in a decrease of intracellular constituents, as it causes the build-up of Reactive Oxygen Species (ROS), higher genomic instability, lower mitochondrial efficiency, and inhibition of ubiquity [5]. Thus, Interference with autophagy disrupts cellular homeostasis and causes the creation of numerous diseases.

Moreover, autophagy plays a complex role in promoting cellular aging and contributing to extended lifespans, depending on the context and specific biological pathways involved [6]. It has been shown in a variety of model species that longevity extension needs autophagy stimulation [7]. This paper presents an overview of autophagy, including its various forms and applications in various disorders.

## 2 METHODOLOGY

All reported data in the present review were assimilated through comprehensive searches of multiple scientific databases, including ScienceDirect (Elsevier), PubMed, Google Scholar, and Google Patents. A combination of keywords was strategically employed to capture relevant studies, including: *‘autophagy’, ‘neurodegeneration’, ‘Alzheimer’s disease’, ‘Parkinson’s disease’, ‘protein aggregation’, ‘lysosomal dysfunction’, and ‘neuronal survival’.* Article selection was according to search keywords observed in the abstract and title. Irrelevant and duplicate articles were excluded, and manual screening of articles was done. Articles conforming to the inclusion criteria were included in the article such as original publication in a peer-reviewed journal. After filtration and screening, 121 published studies that met the inclusion criteria were added to the review. Examination of all the references of identified articles was accomplished, as well as added to the review.

## 3 AUTOPHAGY PROCESS

**Fig. (1):**
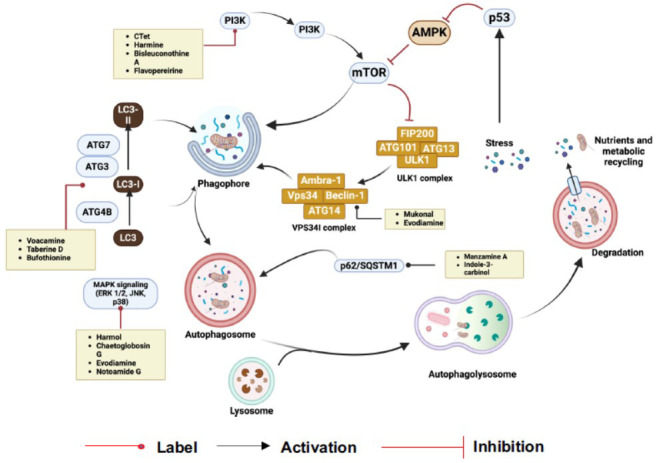
An outline of the autophagy mechanism.

Autophagy is a breakdown phenomenon that was originally transmitted by evolution inside cells. Mammals exhibit three distinct forms of autophagic processes mediated by chaperones, which are collectively referred to as autophagy. Autophagosomes are specific to autophagy and are double-membraned vesicles that capture and incorporate damaged organelles and misfolded or excess proteins from within the cell. These substances are subsequently transferred into lysosomes for deterioration. The mammalian target of rapamycin complex 1 (mTORC1) serves a vital purpose by regulating autophagy—primarily by inhibiting it to maintain a minimal baseline level in almost all cells—thereby contributing to the preservation of cellular homeostasis. When cells experience different kinds of stress, like starvation, withdrawal of growth factors, or hypoxia, mTORC1 inhibition releases autophagy and is greatly increased to fulfill high energy requirements (Fig. **1**).

### 3.1 Initiation of Autophagy

Various cellular stress signals, including oxidative stress, food shortage, or damage to cellular components, trigger the highly regulated process of autophagy. Usually, it begins with activating important signaling pathways, such as the mTORC1 pathway, which prevents autophagy in the presence of nutrients. It is activated as a result of mTORC1 activity being reduced in response to stress signals or a shortage of food [8, 9]. A phagophore, a tiny, double-membrane structure, is created throughout the process and starts to absorb cytoplasmic debris, such as broken organelles or misfolded proteins. After that, the phagophore grows and develops into an autophagosome, which joins forces with a lysosome to break down its contents. Important proteins that start the phagophore's development and encourage autophagy include ATG (autophagy-related proteins) and ULK1 (Unc-51-like autophagy activating kinase 1) [10].

The class III phosphoinositide 3-kinase (PI3K) VPS34 complex, which includes ATG14L, Beclin-1, VPS15, and VPS34, is a downstream autophagy initiation complex that gets phosphorylated by the active ULK1 complex [11]. Phosphatidylinositol 3-phosphate (PI3P) is produced by the VPS34 complex in specific phospholipid membranes, including those at the interfaces of the Endoplasmic Reticulum (ER), ER-mitochondria, and ER-plasma membrane. Omegasomes, which are membrane structures rich in PI3P, are enclosed by PI3P-binding proteins such as zinc finger FYVE domain-containing protein 1 (ZFYVE1/DFCP1) and WD-repeat domain phosphoinositide-interacting proteins (WIPIs). These proteins then interact with other autophagy-related proteins to form the structure of the phagophore [12].

The VPS34 and ULK1 complex are phosphorylated successively through mTORC1 and AMPK in response to cellular stressors. In lipid-enriched membranes, the VPS34 complex produces PI3P, which attracts PI3P-binding proteins. This lipid-protein combination renders it simpler for autophagy-related proteins to assemble themselves, resulting in omegasomes. During the formation of omegasomes into autophagosomes, ATG proteins utilize ubiquitin-like conjugation mechanisms to transform LC3 to LC3-II by binding PE. Meanwhile, adaptor proteins as well as autophagic receptors facilitate the delivery of cargo to the autophagosomes. A dynein-dynactin complex carries the mature autophagosomes close to the lysosomes, where the SNARE complex enables them to become autolysosomes. Lysosomal proteases break down the payloads following the fusion. In the meantime, PI (4,5) P2 creates the proto-lysosomes needed to keep the cytosolic free-lysosome pool intact by creating lysosomal tubule formation sites.

### 3.2 Autophagosome Formation

Two ubiquitin-like conjugation processes involving ATG8s are essential for the expansion of the phagophore. In the initial pathway, the ubiquitin-like activation process, controlled by ATG7 and ATG10, facilitates the ATG12 to ATG5 covalent attachment [13]. Through the coordination of coiled-coil domains, ATG16L1 non-covalently interacts with ATG5 to generate the ATG12-ATG5-ATG16L1 complex, which functions on the phagophore in a manner analogous to an E3 ubiquitin ligase [14]. In the next pathway, the protease ATG4 splits the C-terminal portion of LC3, liberating LC3-I into the cytoplasm [15]. Subsequently, ATG7, ATG3, and the ATG12-ATG5-ATG16L1 complex interact with LC3-I to transform it into LC3-II, which undergoes conjugation with phosphatidylethanolamine (PE). During the elongation stage, LC3-II is closely linked with the double-membrane phagophore [16]. ATG9A is regarded as the major transmembrane protein that has an impact on the core autophagy machinery. Upon autophagy activation, ATG9A is transported to omegasomes from endocytic compartments or the Trans-Golgi Network (TGN), a process that relies on retromer complexes or ULK1 [17]. Because ATG9A-embedding single-membrane vesicles translocate across the autophagosome's exterior membrane, they are important for autophagosome formation; this suggests that the vesicles function as donors of lipid bilayers during the autophagosome formation process [18]. Since ATG9A-containing single-membrane vesicles move across the external membrane of the autophagosome, they play a critical role in autophagosome formation, likely contributing to lipid bilayers during the process [19]. Additionally, ATG18 binds PI3P, linking the phagophore to ATG2, another autophagy-related protein [20]. The ATG2-ATG18 complex is crucial for the binding of PI3P-enriched membranes to the phagophore and for regulating the dimensions of the initial autophagic structures. Evidence has also shown that the yeast Atg2 protein is involved in lipid transport, a key step in autophagy. Increasing evidence depicts that ATG2, VPS21, and the Endosomal Sorting Complexes Required For Transport (ESCRT) complex provide significant roles in autophagosome closure, although the exact mechanisms remain unclear [21].

### 3.3 Autophagosome Maturation

Autophagosomes travel towards lysosomes *via* intracellular trafficking networks to combine with them. Autophagosome migration to areas with lysosome concentrations requires Rab proteins on microtubules and the dynein-dynactin motor complex [22]. The majority of autophagosomes, especially in neurons, are generated at the axon's terminal and are delivered by dynein retrograde to the soma [23, 24]. In the meantime, lysosomes move inside the GTPase- or retromer-dependent way in the direction of autophagosomes. Last but not least, Autophagosomes may combine with lysosomes to create autolysosomes due to the soluble N-ethylmaleimide-sensitive-factor attachment protein receptor (SNARE) complex [25]. The process by which clathrin-mediated lysosomal tube structures develop further out of autolysosomes after the completion of autophagy is known as Autophagic-Lysosomal Reformation (ALR) [26]. PI P2 is formed through an enzyme called phosphatidylinositol-4-phosphate 5-kinase (PIP5K), and it must exist for the start of the tubulation of autolysosomes [27]. The large GTPase dynamin 2 additionally helps with the division of lysosomal tubules within ALR [28].

### 3.4 Selective Autophagy

**Fig. (2):**
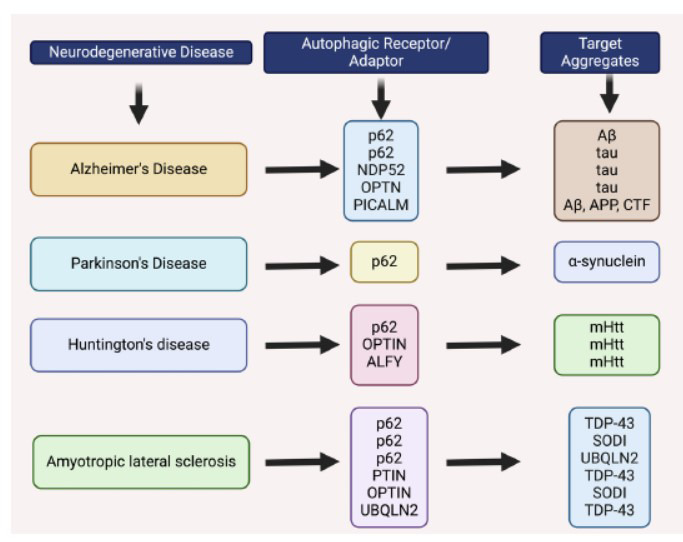
Different autophagic receptors and neurodegenerative disorders.

In the beginning, autophagy was considered to operate as a mass, broad-spectrum breakdown mechanism [29]. However, because so many different kinds of autophagic receptors were found, most autophagy is now thought to be a method of selective elimination [30]. Certain cargoes are recognized and drawn to the autophagosome by autophagic receptors; for instance, p62 recognizes and attracts aggregation of proteins, while ribosomes are recognized by Nuclear FMR1-Interacting Protein 1 (NUFIP1). According to the target molecules, there are various categories of selective autophagy: Agglutination in protein agglomerates proteaphagy for proteasomes, lysophagy for lysosomes, lipophagy for liposomes, granulophagy for granules, pexophagy for peroxisomes, ribophagy for ribosomes, and xenophagy for pathogens [31]. Target molecules are primarily recognized by autophagic receptors *via* certain regions of their conjugated poly-ubiquitin chains, like the UBA domain on p62 or NBR1 and the Zinc Finger (ZF) domain on OPTN [32, 33]. Following this identification, the autophagic receptors directly engage with ATG8s *via* the GABARAP interaction motif (GIM) or the LC3-Interacting Region (LIR) to transport cargos to autophagosomes [34]. A growing body of research has linked different neurodegenerative illnesses to variations in the autophagic receptor gene. Given that most neurodegenerative diseases exhibit protein aggregation accumulation, it is conceivable that autophagic receptor dysfunction— as opposed to additional autophagy-associated proteins— may play an important role in the pathophysiology of these medical conditions (Fig. **2**).

## 4 AUTOPHAGY AND APOPTOSIS

The dual role of autophagy in both cell survival and cell death remains a topic of debate. The observation of autophagy within perishing cells suggests the existence of autophagic cell death. Unlike apoptosis, regarded as type 1 cell death, this is type 2 programmed cell death, which varies morphologically [35]. While caspases mediate apoptosis, a high number of autophagosomes in the cytoplasm mediate autophagic cell death [36]. Normal circumstances result in autophagy and apoptosis suppressing one another, making them dormant [37]. Stress on the mitochondria causes them to produce cytochrome C, which causes apoptosis; on the other hand, stress on the endoplasmic reticulum or changed metabolism (nutrient shortage) may lead to autophagy. In particular, when there are no nutrients available, autophagy promotes survival, while excessive autophagy leads to autophagic cell death. TRAIL (tumor necrosis factor-related apoptosis-inducing ligand), FADD (fasciologenesis-associated protein with death domain), and TNF (Tumour Necrosis Factor) are a few pro-apoptotic signals that can also trigger autophagy. Conversely, pro-survival cues inhibit autophagy *via* another mechanism [38]. Autophagy and apoptosis interact with the family of Bcl-2 protein, the transcriptional factor p53, and atg5 proteolysis. Beclin 1 undergoes interaction with the BH3 domain present in Bcl-2 proteins to control autophagy. Throughout the endoplasmic reticulum, Bcl-2 predominantly serves as an anti-apoptotic molecule; however, it may also function in the form of a mediator that opposes autophagy. The last step is accomplished by blocking Beclin 1 from getting involved with autophagosome nucleation [39]. Additionally, caspases 3-7-8 rupture Beclin 1 to produce N- and C-C-terminal segments that are no longer able to trigger autophagy. C-terminal pieces cause cells to become more vulnerable to apoptotic signals by translocating within mitochondria [40].

Another connection between autophagy and apoptosis is Atg5. It correlates with the apoptosis-promoting FADD protein to induce autophagy-associated cell death [41]. After Atg5 is cleaved by Calpain inside the N-terminus, the segments enter the mitochondria and communicate with Bcl-xL to trigger caspase activation and cytochrome C release, which ultimately leads to apoptosis [42]. In neonatal hypoxia-ischemia, dying neurons exhibit concurrent apoptotic and autophagic characteristics [43]. A comprehensive investigation of the molecular processes underlying autophagy and apoptosis will enhance comprehension of their interaction and could potentially uncover strategies for modifying them in neurodegenerative disorders.

## 5 SELECTIVE AND NON-SELECTIVE AUTOPHAGY AND PHYSIOLOGICAL FUNCTIONS IN RELATION TO HEALTH

It is demonstrated that autophagy acts selectively under specific circumstances, even though it was believed to be a non-selective breakdown process earlier. Whereas cargo labeling and adaptor proteins inside autophagosomes determine selectivity during selective autophagy, non-selective autophagy simultaneously breaks down cytoplasmic constituents [44]. The class of proteins known as adaptor proteins, which bind to cargo, includes the gamma-aminobutyric acid (LC3) protein, a class of autophagosome-localized proteins known as receptor-associated protein (GABARAP); furthermore, the distribution of adaptor proteins to payload or the ubiquity of cargoes identifies them. One common autophagy receptor that determines the autophagy preference is p62/Sqstm1 (p62). P62 serves as a mediator between ubiquitin signaling and selective autophagy [45]. Phosphorylated p62 ensnared the invasive bacteria and damaged mitochondria as they arose. Increased mTORC1 phosphorylation of mouse and human Ser351/Ser349 results in a higher affinity across Keap1 and p62. Phosphorylated p62 attached to Keap1 undergoes interaction with LC3 *via* the LC3-Interacting Region (LIR) to be broken down in the autophagy pathway [46].

### 5.1 Mitophagy

One process called “mitophagy” involves the targeted destruction of damaged mitochondria. Mitochondria is important for ATP synthesis, the generation of phospholipids, the activation of apoptosis, and the provision of energy to the cell. When electrons *via* the electron transport chain escape from mitochondria and interact with the surrounding oxygen, reactive oxygen species (ROS) are created [47]. Tumor initiation is linked to the build-up of mitochondrial breakdown and the reactive oxygen species production [48]. Therefore, by effectively eliminating abnormal mitochondria, mitophagy helps in the prevention of tumor growth. Indeed, the deletion of Bcl-2 adenovirus E1B 19 kDa-interacting protein 3 (BNip3), a hypoxia-inducible protein that tends to target mitochondria, causes a defect in mitophagy, which accelerates the evolution of mammary tumors [49]. In its faulty forms, the PINK1/parkin complex, which is generated by phosphatase and tensin homolog (PTEN), is believed to be a major cause of familial Parkinson's disease (PD). It also phosphorylates ubiquitin and serves as a marker for mitochondria during mitophagy [50].

### 5.2 Allophagy

It is known that maternal inheritance occurs for mitochondrial DNA (mtDNA) in a variety of organisms. The precise process of mtDNA inheritance is yet unknown, though. According to a new study on Caenorhabditis elegans, after fertilization, when the autophagy receptors IKKE-1 and ALLO-1 become phosphorylated throughout the selective autophagy procedure, paternal organelles are marked by ubiquitin and eliminated [51]. For Drosophila, mice, and C. elegans, preferred autophagy targeting paternal mitochondria can be observed [52]. As a result, it is thought to be vital for understanding the maternal inheritance method.

### 5.3 ER-Phagy

Depending on whether surface ribosomes are present or absent, the ER is classified as having smooth or rough surfaces. It contributes to calcium ion buildup, drug metabolism, lipid synthesis, protein folding, and transport [53]. In particular, it has long been hypothesized that drug metabolism-related ER deformity degradation involves autophagy. An ER-phagy receptor that undergoes interaction with LC3 and GABARAP belongs to the FAM134 reticulon protein family, as demonstrated by Khaminets *et al*. Mice deficient in the FAM134B ER-phagy receptors show peripheral neuropathy as a clinical manifestation and ER dilatation [54]. Furthermore, it has been noted that a variety of malignant tumors have mutations or alterations in FAM134B expression [55]. These results suggest that ER-phagy has an important part in both malignancies development and peripheral nerve homeostasis.

### 5.4 Lysophagy

Numerous hydrolytic enzymes found in lysosomes are crucial for intracellular digestion. The cell can use ESCRT machinery to repair lysosomes when damage to them is minimal [56]. If this repair is unsuccessful, ubiquitin-tagged lysosomes are cleared through selective macroautophagy, a phenomenon called lysophagy. The regulation of lysosome quality requires lysophagy. Lysosomal damage is brought on by hyperuricemia, tau protein, amyloid-beta protein, Huntingtin protein, and type 2 diabetes. Additionally, there have been findings linking a decrease in lysophagy to diseases connected to lifestyle, including neurodegenerative disorders [57].

### 5.5 Nucleophagy

One of the most significant organelles in a cell is the nucleus, which also houses the transcriptional site and the genetic material of the cell. There are two types of nucleophagy: macro and micronucleophagic. Specifically, it was suggested that autophagy's selective nuclear lamina destruction could prevent the growth of tumors [58].

### 5.6 Pexophagy

Organelles called peroxisomes are engaged in various metabolic processes, like the formation of phospholipids, bile acids, purine catabolism, along long-chain fatty acid beta-oxidation. As a result of fatty acid β-oxidation, these self-replicating organelles produce Reactive Oxygen Species (ROS). Pexophagy, which prevents oxidative damage, illnesses including cancer, along with abnormalities in peroxisome biosynthesis, helps cells maintain peroxisome homeostasis [59]. Pexophagy is a catabolic mechanism that enables autophagy to degrade peroxisomes specifically. Peroxisome membrane proteins, like PEX3 and PEX5, have been demonstrated to be significant regulators of pexophagy [60].

### 5.7 Lipophagy

Phospholipids envelop the lipid ester mass, primarily consisting of cholesterol and triglycerides, to form lipid droplets. For the body to continue producing energy, lipids must accumulate and be used by the cell. When cellular lipids undergo digestion, fatty acids emerge from the triglycerides, and they accumulate in lipid droplets during energy-deficient conditions (hunger). The mechanism that breaks down lipid droplets specifically is called lipophagy [61]. Higher triglycerides and lipid droplet levels inside hepatocytes of mice lacking the Atg7 gene indicate a significant function of lipophagy as a mechanism for lipolysis in living organisms. It was found by Takahashi *et al*. that oval cells could have differentiated into hepatocytes by releasing Atg7, rather than Atg7-deficient hepatocytes, which were responsible for the fat buildup in mice's livers that lacked Atg7 [62]. This is a major discovery that illuminates the connection between autophagy and fat metabolism.

### 5.8 Xenophagy

To proliferate, different types of cells allow bacteria and viruses to enter their cytoplasm. The autophagosome recognizes intracellular pathogens as foreign materials by a process called xenophagy, differentiates them, and then delivers them into the lysosome for breakdown and removal. When a bacterial infection results in membrane damage, ubiquitination sets off xenophagy. However, other bacteria, including Legionella pneumophila, prevent autophagy by preventing the development of phagophores [63].

### 5.9 Aggrephagy

Even when the separation membrane or autophagosome forms normally, anomalies can arise during the autophagosome's integration of its substrate molecules, causing abnormal autophagosomes and clusters to pile up inside the cell. Inflammation, reactive stress-related neurotoxicity, irregular calcium homeostasis, inadequate neuronal permeability of membranes, and physiological abnormalities are all brought on by aberrant protein aggregation [64]. Aggrephagy is the word used to describe the process by which autophagy preferentially breaks down aggregated proteins.

### 5.10 Ribophagy

The ribosome converts information from mRNA into polypeptides, which is how it carries out protein synthesis *in vivo*. According to Kraft *et al*., ribophagy is the term given to the selective autophagy process that causes ribosomes to be preferentially broken down in nitrogen-starved budding yeast. Ribophagy efficiently inhibits new protein synthesis and recycles resources during hunger. According to Wyant *et al*., ribosomal selective autophagy is mediated by nuclear fragile X mental retardation-interacting protein 1 (NUFIP1) [65].

### 5.11 Nuclear Pore Complex (NPC)-Phagy

NPC is regarded as a big structure of a protein that is enclosed within the nuclear envelope. Besides mediating movement between the cytoplasm as well as the nucleus, the NPC is crucial for the expression of certain genes. The ability of the protein complexes inside the organelle to perform selective autophagy is still unknown. 2020 saw the demonstration by two research groups of receptor-dependent selective autophagy as the means of NPC degradation [66]. NPC-phagy improves the overall regulation of NPC quantity and quality. It is yet unknown, nevertheless, how NPC-phagy functions in illness.

### 5.12 RN/DN-Autophagy

“Following their delivery into the lysosome, nucleic acids undergo intrinsic digestion *via* the RN/DN-Autophagy (RDA) mechanism. Through these routes, DNA and RNA are picked up in the lysosome and immediately degraded there, respectively. Lysosome-Associated Membrane Protein (LAMP) 2C is a lysosomal membrane protein that serves as a receptor by binding to RNA and DNA [67]. In RDA, it is discovered that a transmembrane protein called the SID1 transmembrane family, member 2 (SIDT2) acts as a nucleic acid transporter within the lysosomal membrane. Although precise processes for RDA are yet unknown. According to Tan *et al*., RNautophagy and SIDT2 had an impact on the growth of pulmonary and gastrointestinal tumors in mice [68].

### 5.13 Neuronal Autophagy

It has been noted that the autophagic mechanism generally shields the neurons. Synaptic plasticity, glial cell anti-inflammatory activity, oligodendrocyte growth, and the myelination process all depend on neuronal autophagy [69]. Although neurons are post-mitotic cells, their aggregate-prone proteins are unable to be diffused *via* cell division. Thus, neurons need systems that manage the quality control of proteins. Therefore, aberrant proteins accumulate as a result of altered protein degradation system activity, which ultimately results in neuronal dysfunction like dysregulated transcription and reduced axonal transmission [70].

## 6 RELATIONSHIP OF AUTOPHAGY PATHWAY WITH PATHOGENESIS OF NEURODEGENERATIVE DISEASES

Neurodegenerative diseases are characterized by pathologically atypical aggregates of proteins that grow into neurofibrillary tangles [71]. The autophagy-lysosome degradation process is the primary target of this protein aggregation observed in neurodegenerative diseases. Likewise, autophagic receptor genetic mutations, including those in p62, ALFY/WDFY3, OPTN, and NBR1, have frequently been linked to neurodegenerative illnesses [72]. The most prevalent neurodegenerative risk factor, aging, also markedly reduces autophagic activity. Thus, it is believed that neurodegenerative disorders may arise as a result of impaired autophagy. A recent study indicates that regulating autophagy may present a viable approach to managing these ailments. Autophagy activation boosted the removal of proteins such as mHtt, insoluble tau, and Aβ42 that are prone to aggregation, and this clearance was dependent on the p62 aggrephagy receptor [73]. On the contrary, mHtt aggregates were elevated inside rat brains and cell culture systems when bafilomycin A1 (Baf. A1) or 3-MA suppressed autophagy [74]. An increasing variety of neurological conditions, such as Alzheimer's disease (AD), Amyotrophic Lateral Sclerosis (ALS), Parkinson's Disease (PD), Huntington's Disease (HD), and multiple sclerosis (MS), have been linked with autophagy.

### 6.1 Regulation of Autophagy in Alzheimer’s Disease (AD)

AD is a kind of progressive dementia that is most prevalent. Through the initial phases, the infected person has issues like recalling current occurrences. Anxiety, confusion, rage, mood fluctuations, and difficulties in writing and interpreting are all indicators that the illness is growing worse [75]. Two anomalous formations, intra-neuronal fibrillary tangles and aging plaques are indicative of Alzheimer's disease. The majority of the deadly protein peptides that make up amyloid plaques are insoluble and referred to as beta-amyloid peptides (A). These peptides are produced when an enzyme cleaves the bigger protein known as amyloid precursor protein (APP). Tau, a microtubule-associated protein, is found in highly phosphorylated forms in neurofibrillary tangles. Tau can work with tubulin to regulate vesicle trafficking in neurons and stabilize microtubules. This protein possesses the ability to aggregate into two helical filaments in pairs that form tangles in neurofibrillation when tau kinases improperly hyperphosphorylate it [76].

The initial indication that autophagy could be a factor in the pathophysiology of AD was provided by the identification of various autophagic vacuoles (AVs) inside AD brains. Immune gold labelling as well as electron imaging revealed a remarkable build-up of juvenile AVs in neurites with dystrophies [77]. APP and beta-cleaved APP were present in the purified AVs. Together with A, these vacuoles had significant levels of presenilin-1 (PS1) and nicastrin enrichment. In addition to this finding, other data point to potential impairments in autophagosome-lysosome fusion and AV transport in AD. Because autophagosomes transit retrogradely up the axon, which is necessary for neurons' autophagosomes to fuse with lysosomes, defective autophagosome retrograde transport causes immature AVs and Aβ-generating AVs to accumulate, which increases Aβ generation [78]. Furthermore, it was demonstrated that A controls autophagy by AMPK activation-induced AV formation and lipid raft-localized BECN1-induced autophagy [79]. An intriguing study demonstrates a gamma-secretase-independent role of PS1 in the lysosome. Although PS1 is an essential component of the APP-cleaving gamma-secretase complex, Lee *et al*. (2010) showed that mutations in PS1 impair lysosomal function and promote neuronal death. In PS1-null blastocysts, lysosomal dysfunction arises due to the absence of PS1-dependent targeting of the v-ATPase V0a1 subunit to lysosomes, which in turn inhibits substrate proteolysis and autophagosome clearance. The elimination of soluble and insoluble monomeric and oligomeric tau clumps is considered to be assisted by autophagy in UPS. Chloroquine specifically inhibits autophagosome-lysosome fusion, which delays tau clearance and culminates in the accumulation of tau aggregates [80].

The autophagy is essential for Aβ accumulation along with neurofibrillary tangles. Novel discoveries that might accelerate the breakdown of harmful aggregates are crucial for effective therapy, as abnormal accumulation of proteins is the primary etiology of most neurodegenerative illnesses. Therefore, autophagy is considered as an emerging therapeutic target because it is primarily in charge of breaking down aberrant proteins or organelles. Additionally, autophagy provides a defence against apoptotic insults and a variety of stressors [81]. Finding triggers for autophagy can therefore be a useful therapeutic approach. Non-toxic small compounds have been examined for their ability to repair autophagy in neurons at the basic level. Significant medical advantages have been demonstrated with compounds that induce autophagy in numerous animal models of neurodegenerative conditions. In an AD mouse model, the selective inhibitor of TORC1, rapamycin, improves the pathology caused by tau and Aβ [82]. Dimebon, another name for latrepirdine, enhances autophagy in the mouse brain that is reliant on Atg5 and decreases a neurological condition [83]. Metformin, a protein phosphatase 2A (PP2A) agonist, is currently being evaluated in clinical studies for AD. It suppresses tau hyperphosphorylation by blocking TORC1 [84]. Furthermore, SMER28—a small-molecule enhancer of rapamycin—significantly reduces Aβ peptide and APP-CTF levels in a manner that is independent of gamma-secretase [85]. AMPK, a ULK1 kinase, can also be activated for the induction of autophagy in a way that is TORC1-independent. Lithium has been linked to a reduction in AD pathogenesis through AMPK activation and autophagy modulation [86]. AMPK activation is one of the numerous molecular activities of resveratrol and its analogs, RSVA314 and RSVA405, which also exhibit protective effects against AD [87]. In an AD mouse model, nicotinamide reduces autophagosome accumulation *via* improving lysosome/autophagosome acidification, hence preventing pathology and cognitive loss [88].

BECN1 mimetics and virally packaged BECN1 may additionally prevent the development of harmful complexes by focusing on autophagy’s initial stages, despite the lack of pathologic evidence in AD [89]. It seems that by breaking down these aggregates, autophagy-inducing substances can stop tangles and plaques from developing in the initial phases of AD. Nevertheless, in advanced AD stages, when a lysosomal function or autophagosome/lysosome fusion is compromised, autophagy stimulation may worsen the disease [90]. Thus, for improved AD therapy, innovative methods that enhance lysosomal and autophagosome fusion activity would be crucial.

### 6.2 Regulation of Autophagy in Parkinson’s Disease (PD)

Parkinson's disease (PD) is regarded as a long-term, degenerative movement illness that is regulated through autophagy. Progressive neurodegenerative movement disorder Parkinson's disease (PD) is marked by α-synuclein gene proliferation and aberrant α-synuclein aggregates called Lewy bodies found in the substantia nigra's dopaminergic neurons. It has been shown that ATG7 knockout causes age-dependent increases in α-synuclein inclusion bodies containing p62 in dopaminergic neurons as well as impairments in motor performance in elderly mice [91]. Furthermore, the autophagy-lysosome system degrades α-synuclein that carries harmful mutations. On the other hand, inclusions of α-synuclein hinder the autophagic process across multiple stages. For example, it was shown that inclusions of α-synucleins inhibit the development of omegasomes by causing ATG9A to become mislocalized [92]. Moreover, α-synuclein aggregates harm autophagosome retrograde transport, but autophagosome-lysosome fusion is unaffected. Finally, α-synuclein disrupts the process of autophagic clearance and the function of lysosomal aspartyl protease cathepsin D (CTSD) [93]. For Parkinson's disease, transcriptional changes to genes associated with autophagy are commonly seen. In contrast to the pre-symptomatic stage, TFEB-mediated transcription of Beclin-1, CTSD, and LAMP1 is decreased during the Parkinson's disease (PD) symptomatic stage when a large number of dopaminergic neurons within the midbrain are currently impaired [94]. Additionally, zinc finger protein, a transcriptional repressor containing KRAB and SCAN domains 3, which controls p62 and LC3 transcription, underwent a rise in nuclear translocation upon the expression of human A30P α-synuclein. As expected, upregulation of TFEB improved the clinical characteristics of Parkinson's disease (PD), particularly α-synuclein accumulation, motor dysfunction, and midbrain neurodegeneration [95].

The primary cause of Parkinson's disease (PD) is mutations affecting the lysosomal enzyme glucocerebrosidase (GBA), which breaks down glucosylceramide. The PD-associated GBA mutations (N370S and L444P) lower the concentrations of protein as well as enzyme function, thus preventing it from migrating from the ER towards the lysosomes. This causes target lipids to accumulate in lysosomes and causes ER stress, which ultimately causes lysosomal dysfunction and autophagy [96]. Initially, individuals with sporadic PD possess increased α-synuclein inclusions and a selective decline in GBA activity. The buildup of α-synuclein oligomers is facilitated by either direct inhibition or the N370S mutation of GBA. Moreover, it has been demonstrated that the GBA target lipid glucocerebroside can stimulate the development of α-synuclein fibrils, which can subsequently bind directly to lysosomal membranes and impede GBA activity and trafficking, aggravating Parkinson's disease further [97]. It is primarily brought on by mutations that affect the leucine-rich repeat kinase 2 (LRRK2/PARK8) gene. Around 40 harmful LRRK2 mutations have been identified in PD patients. Whether LRRK2's function in autophagy is connected with disease pathophysiology is still under debate, though. According to Tong *et al.*, the LRRK2 deletion damages the autophagy-lysosome pathway and causes cell death [98]. A gain-of-function mutation refers to a genetic change that enhances or alters the normal activity of a protein. In the case of LRRK2, mutations like G2019S and R1441C increase the protein's activity, which contributes to disease development, such as Parkinson's disease. Similar to LRRK2 deficiency, the mutations causing LRRK2 to undergo gain-of-function boost the protein's kinase activity but inhibit autophagic breakdown [99].

According to multiple investigations, the LRRK2-G2019S mutation limits endocytic vesicular trafficking by inhibiting small GTPase activity, whereas the LRRK2-R1441C mutant hinders lysosomal activities by improperly connecting with lysosomal v-ATPase. These findings explain the contradiction [100]. The mutations that cause function loss of ATPase cation transporter 13A2 (ATP13A2), related to a Parkinson's disease variation having a rapid onset, have been identified to stay within the extracellular reticulum rather than being moved into the lysosomes. ATP13A2 is a lysosomal type 5 P-type ATPase that is needed to sustain a lysosomal pH. Low concentrations of ATP132A2 proteins were detected in Lewy bodies, while dopaminergic neurons in Parkinson's disease patients had lower levels of ATP13A2 protein. Moreover, it has been observed that PD-associated mutations in ATP13A2 decrease lysosomal acidification [101]. The molecular processes underpinning the ATP13A2-mediated autophagy-lysosome pathway have been revealed by recent investigations. Depletion of ATP13A2 disrupts mTORC1 activity, causing TFEB, a key transcription factor for autophagy-related genes, to be retained in the cytoplasm rather than translocating to the nucleus [102].

Furthermore, lysosomal dysfunction brought on by ATP13A2 deficiency-induced downregulation of Synaptotagmin 11 (SYT11) interferes with the breakdown of autophagy. A separate study shows that ATP13A2 promotes cortactin deacetylation in an HDAC6-dependent manner, which contributes to the development of an F-actin network and thereby enhances autophagosome-lysosome fusion [103]. Moreover, it has been established that a mutation in ATP13A2 promotes α-synuclein accumulation, and that α-synuclein silencing might lessen the neurotoxicity caused by ATP13A2 depletion. These results suggest that α-synuclein aggregation caused by ATP13A2 loss might correlate with the pathophysiology of Parkinson's disease. It has recently been proposed that the VPS35 mutation, a fundamental retromer complex component that controls lysosomal protease trafficking, might be a factor in the abnormal autophagy noticed in Parkinson's disease [104]. The PD substantia nigra was reported to have lower VPS35 mRNA levels. Research has shown that the familial PD-associated VPS35 mutation D620N impacts the endosomes' ability to recruit the WASP and Scar homolog (WASH) complex, which leads to ATG9A mislocalization as well as compromised autophagy.

### 6.3 Regulation of Autophagy in Huntington’s Disease (HD)

HD is an inherited neurological disease that develops over time and results in motion disability, aberrant behavior, and memory loss. Inside the cerebral cortex and striatum, the mHtt proteins that arise from the excessive growth of a polyQ repetition in the first exon form ubiquitin-positive complexes with β-sheet structures that are cytotoxic. Autophagosome accumulation coexists with progressive motor impairments caused by mHtt overexpression. In a similar vein, HD patients showed a buildup of autophagic vacuoles [105]. Numerous investigations have illustrated that aggregate-prone proteins with polyQ expansion are removed by autophagy in both *in vivo* and cell culture systems. In those investigations, mHtt aggregation was elevated by either genetic modification or autophagy blockage through the utilization of autophagy inhibitors like 3-MA or Baf.A1. In contrast, rapamycin or trehalose, two autophagy activators, decreased the number of inclusion bodies. Depending on its structure, the turnover rate of mHtt changed and affected how it interacted using the OPTN and p62 aggrephagy receptors [74]. This abnormal buildup of mHtt as well as HD progression in mouse models was further encouraged *via* the aggrephagy adaptor ALFY heterozygous deficiency, which is accountable for making it simpler for the p62-binding intracellular aggregates to be directed by autophagosomes.

Based on a new genome-wide screening inside the striatum, several genes associated with autophagy, including Atlastin 3, Tfeb, and Atg4b, seem to protect against mHtt toxicity. Normal huntingtin helps different autophagy proteins recognize cargo and inhibits Beclin-1 ubiquitination by acting as a scaffolding protein. Nevertheless, mHtt is unable to identify cytosolic payloads within autophagosomes [106]. It has also been shown that mHtt reduces Beclin-1 function by drawing Beclin-1 to inclusion bodies or by decreasing RASD family member 2 (RASD2/Rhes), a striatal-specific GTPase which serves to distinguish the inhibitory linkages among Beclin-1 and Bcl-2. Furthermore, mHtt overexpression or huntingtin Knockdown in neurons can lead to defective autophagosome retrograde transmission [107].

### 6.4 Regulation of Autophagy in Amyotrophic Lateral Sclerosis (ALS)

Cytoplasmic ubiquitin-positive inclusion development and death of motor neurons that control voluntary muscles are two hallmarks of ALS, an uncommon neurodegenerative illness that progresses slowly. The majority of instances of ALS are random, however, between 5 and 10% are brought on by inherited TDP-43genetic mutations, SOD1, ubiquilin 2, fused in sarcoma (FUS), or TDP-43 (UBQLN2) [108]. Autophagy was proposed as a possible contributor to the aetiology of ALS in multiple studies.

It has been found that improved neuromuscular junction changes and tremors, which are linked to ALS, occur in motor neuron-specific ATG7 deletion mice having a pathogenic mutation in SOD1 [109]. Thirdly, Bcl-2 and aberrant ATG4B protein transcription were enhanced by TDP-43 suppression or one of the mutations linked to ALS, leading to autophagic abnormalities. In the end, activation of autophagy decreased TDP-43 aggregation as well as enhanced the likelihood of survival of human motor neurons with TDP-43 mutations [110]. Autophagy has been linked to UBQLN2, an additional genetic risk factor for ALS in families. When UBQLN2 and LC3 combine, cargo proteins in autophagosomes, such as the autophagy adaptor, are ubiquitinated. Furthermore, the autophagosome-lysosome fusion is facilitated by UBQLN2.

It has been demonstrated that loss of UBQLN2 raises the amounts of ubiquitinated TDP-43 and prevents degradation of autophagy by encouraging lysosomal v-ATPase fragmentation [111]. When an ALS-associated UBQLN2 mutant, like P497H, was overexpressed, UBQLN2 inclusions containing p62 and ubiquitinated protein aggregates accumulated. This led to impaired autophagy and ALS-like symptoms [112]. TDP-43 inclusions were observed to work with p62 and were eliminated by p62 overexpression, as was previously mentioned. Conversely, the increased insoluble protein aggregates brought on by the p62 loss made the symptoms of ALS worse [113]. Importantly, the UBA, LIR, and PB1 areas of p62 include over half of the mutations linked to ALS. These areas interact with either LC3 or cargo proteins, indicating a potential connection between the ALS pathophysiology resulting from p62 mutations and the ineffective transport of protein aggregates to the autophagosomes [114].

Through the region of its coiled-coil, OPTN, another aggrephagy receptor, identifies inclusions in protein aggregates and facilitates their clearance. Necroptosis-dependent axonal degeneration resulted from an increase in TDP-43 or SOD1-containing inclusions induced by OPTN silencing or its ALS-related mutations [115]. OPTN is an aggrephagy receptor, but it also uses MYO6 and TOM1 to facilitate autophagosome trafficking. Patients with ALS or those with mutations frequently experience disruptions in the connection between OPTN and MYO6 [116]. ALS can also be driven by abnormalities inside the ESCRT-III intricate, which is vital for the maturation of autophagosomes. In several model systems, it has been discovered that ALS-related point mutations in CHMP2B, a subunit of the ESCRT-III complex, cause an autophagosome overgrowth and lysosome mislocalization, which form intracellular inclusions [117].

### 6.5 Regulation of Autophagy in Hereditary Spastic Paraplegia (HSP)

Leg weakness and spasticity worsen over time in a variety of hereditary neurodegenerative disorders known as HSPs. These disorders result from the corticospinal motor neurons' axonal degeneration. Up till now, more than 60 genes have been identified and there are now over 80 known genetic loci linked to spastic gait (SPG). Despite the existence of several SPG genes, the molecular etiology of HSPs is limited to a few cellular processes, including lipid metabolism, axonal transport, shape and synthesis of organelles, membrane trafficking, and mitochondrial activity [118]. Autosomal-recessive HSP is usually triggered *via* mutations in the spatacsin gene, SPG11. It has been discovered that aberrant lysosomal homeostasis, which can include autolysosome accumulation and ALR impairment, is caused by spatacsin loss in mouse neurons [119]. Like spatacsin, depletion of the SPG15-encoded spastizin decreased ALR initiation, which in turn decreased the number of free lysosomes and, eventually, the build-up of waste products within the cell. By stimulating Rab-dependent endosome-autophagosome fusion or by engaging by Beclin-1-UVRAG-Rubicon complex, spastizin also controls the maturation of autophagosomes [120].

Subunits of the adaptor protein (AP) complex are encoded by many SPG genes. The AP complex is essential for sorting intracellular cargo between various organelles and is found in the endosome or TGN. SPG50, SPG51, SPG52, and SPG47, appropriately, encode the AP-4 subunits β-1, µ-1, ε-1, and σ-1, which are linked to childhood-onset HSP. When the AP-4 complex is absent, ATG9A-containing vesicles are replaced from the TGN to autophagosome-forming sites inside neurons. This is because the AP-4 complex separates the target proteins from the TGN and transports them to a unique membrane structure [121]. Encoded by SPG48, AP-5 ζ-1 is a member of the AP complex linked to HSP. According to a prior study, AP-5 undergoes interaction with both spastizin as well as spatacsin. The aging AP-5 ζ-1 knockout mice's incorporeal Golgi apparatus and corticospinal tract exhibited deterioration, comparable with phenotypes generated by silencing spatacsin or spastizin. Furthermore, the AP-5 Η-1 knockout animals exhibit abnormalities in autophagic clearance and impairment in ALR [122].

## 7 AUTOPHAGY UPREGULATION AS A THERAPEUTIC STRATEGY FOR NEURODEGENERATIVE DISEASES

**Fig. (3):**
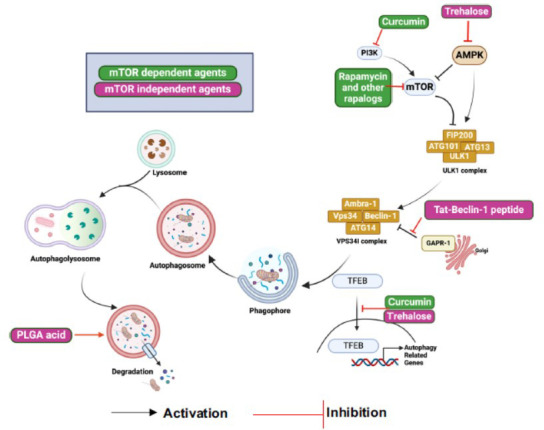
Mechanisms of action of drugs that induce autophagy.

The pathophysiology of hereditary neurodegenerative illnesses is associated with a variety of genetic abnormalities that decrease autophagic activity, and these mutations seem to exacerbate the disorders. Autophagy upregulation can help delay the progression of ALS, PD, HD, and AD by reducing the accumulation of proteins prone to intracellular aggregates. This implies that a great deal of neurological conditions may be treated with autophagy induction (Fig. **3**) [123].

The aggregate-prone protein removal is improved by treatment with different autophagy-inducing drugs, either mTOR-dependently or-independently. Rapamycin and curcumin, two mTOR-dependent inducers of autophagy, directly block mTORC1 activity, which activates the ULK1 complex. On the other hand, drugs that do not rely on mTOR stimulate autophagy activity using different intracellular signaling pathways or lysosome biogenesis.

Nevertheless, maintaining cellular homeostasis is damaged by prolonged autophagy. Therefore, care should be used when focusing on autophagy as an option for therapy for neurodegenerative illnesses.

### 7.1 mTOR-Dependent Autophagy Inducing Agents

Autophagy can be induced by two different kinds of small molecules, either mTOR-dependently or -independently. Rapamycin is the first known inducer of mTOR-dependent autophagy. Through allosteric binding, rapamycin suppresses the kinase activity of mTORC1. In experimental settings, although rapamycin has not been authorized for therapeutic usage, it tends to decrease neural demise as well as alleviate a great deal of neurodegenerative disease symptoms by activating autophagy [124]. Owing to rapamycin's restricted absorption, its derivatives, sometimes known as rapalogs, like Everolimus (RAD001), ridaforolimus (AP23575), and temsirolimus (CCI-779). One of these, everolimus, was currently approved through Food and Drug Administration (FDA) to treat tuberous sclerosis. Apart from rapamycin, curcumin is a naturally occurring substance that has been reported to have a healing impact on neurodegenerative diseases *via* suppression of the PI3K/Akt/mTOR pathway [125]. Curcumin undergoes demonstration for stimulating the synthesis of autophagy-related proteins like ATG16L1, ATG5, and Beclin-1 in addition to motor proteins required to transfer axons retrogradely. This increases autophagic flux and helps to remove intracellular aggregates [126].

### 7.2 mTOR-Independent Autophagy-inducing Agents

Extended usage of mTOR-dependent autophagy inducers may lead to adverse effects, as TOR signaling performs a variety of autophagy-independent tasks, including ribosome synthesis. Thus, inducers of autophagy that are not dependent on mTOR have been created. Trehalose is a typical mTOR-independent inducer of autophagy that increases autophagy by activating TFEB or AMPK [127]. According to several studies, this disaccharide reduces neuronal mortality and prevents the creation of protein aggregates, which slows the course of neurodegenerative diseases. Certain mood stabilizers, including loperamide, clonidine, verapamil, and calpastatin, impede the autophagosome formation process by lowering inositol phosphate 3 (IP3) levels, which in turn causes the autophagy-lysosome system to degrade aggregate-prone proteins. Lithium inhibits inositol monophosphatase (IMPase) in the phosphoinositol cycle, while valproic acid and carbamazepine decrease inositol production [128, 129]. Autophagosome accumulation brought on by faulty lysosomal ATPase-induced lysosomal alkalization inhibits the degradative process. Thus, the best course of action for enhancing autophagy may be to re-acidify lysosomes. Poly (DL-lactide-co-glycolide) (PLGA) acidic nanoparticles have undergone demonstration for restoring lysosomal pH, hence rescuing lysosomal deficiencies and autophagic degradation [111].

## CONCLUSION

While mutations in different genes cause hereditary neurodegenerative disorders, one feature they all have in common is the build-up of protein aggregates. Therefore, proteinopathies include neurodegenerative disorders. Several studies showed that the intracellular protein aggregates can interfere with multiple stages of autophagy and that multiple genes linked to these circumstances are implicated in the autophagy-lysosome pathway. Therefore, we hypothesize that upregulating autophagy may help treat neurodegenerative illnesses. Autophagy activation alleviates neurodegenerative symptoms and decreases inclusion body formation in a variety of experimental setups. Nevertheless, the use of autophagy inducers as an intervention for neurodegenerative disorders is still relatively new. Most current pharmacological techniques for modulating autophagy are predicated on inducing autophagy in its entirety. Besides, harmful effects arise from the over-activation of autophagy. It is important to use targeted strategies to address particular autophagy stages. Therefore, a detailed understanding of autophagy's roles in different neurodegenerative disorders is necessary (Table **1**).

## STUDY LIMITATIONS

Although this study provides an overall description of how autophagy contributes to the occurrence of the condition of a neurodegenerative, it can be restricted to a number of limitations. First, the review is limited to secondary data available in the literature so it can be biased because of the selectivity or may be a biased because of the dynamic nature of research in this fast developing area. Most of the studies already mentioned are either preclinical or animal based and would be directly untranslatable to human physiology and pathology. Moreover, excessive use of autophagy as a specialized skill increases its complexity and the current state of autophagy dependency necessitates the difficulty of defining clear causality connections between impaired autophagy and certain neurodegenerative phenomena. The limitation to assess long-term effects of autophagy modulation is also due to the absence of longitudinal clinical data. Lastly, though the therapeutic potential is addressed, there is still a lot of ground to cover in clinical application of autophagy-inducing agents, as a lot of these compounds are not subjected to rigorous human testing. Refinements in the future can be done to tame these limitations by focusing on the mechanistic investigation in human models and standardized clinical trials to prove the therapeutic efficacy.

## AUTHORS’ CONTRIBUTIONS

The authors confirm their contribution to the paper as follows: BB: Writing the draft, MD: Writing, reviewing and conceptualization, G: Figures drawing, data collection, KW: Data collection and review, SG: Review and experts' comments and SC: Review. All authors reviewed the results and approved the final version of the manuscript.

## Figures and Tables

**Table 1:** List of clinical trials for autophagy-related diseases.

**Trial**	**Disease**	**NCT Number**
Study of Autophagy and the effects of GALIG gene products in HIV-1 infected patients who are under antiretroviral therapy since primary infection, chronic phase, or never treated	HIV Infections	NCT04160455
MEK and autophagy inhibition in metastatic/locally advanced, unresectable neuroblastoma RAS (NRAS) Melanoma (CHLOROTRAMMEL)	Melanoma	NCT03979651
Study of combination therapy with the MEK inhibitor, Cobimetinib, Immune checkpoint blockade, Atezolizumab, and the autophagy inhibitor, hydroxychloroquine, in KRAS-mutated advanced malignancies	Gastrointestinal cancer	NCT04214418
Sirolimus or Vorinostat hydroxychloroquine in advanced cancer	Advanced cancers	NCT01266057
MLN9708 and Vorinostat in patients with advanced p53 mutant malignancies	Advanced cancers	NCT02042989
LY3214996 +/- HCQ in pancreatic cancer	Pancreatic cancer Advanced cancer	NCT04386057
Combined cafilzomib and hydroxychloroquine in patients with relapsed/refractory multiple myeloma	Multiple Myeloma	NCT04163107
Catalyzing the containment of COVID-19	COVID-19	NCT04523090

## LIST OF ABBREVIATIONS

AD: Alzheimer's Disease
ALR: Autophagic-lysosomal Reformation
ALS: Amyotrophic Lateral Sclerosis
ATG: Autophagy-related Proteins
AVs: Autophagic Vacuoles
ESCRT: Endosomal Sorting Complexes Required for Transport
FADD: Fasciologenesis-associated Protein with Death Domain
GBA: Glucocerebrosidase
HD: Huntington's Disease
LAMP: Lysosome-associated Membrane Protein
MS: Multiple Sclerosis
mTORC1: Mammalian Target of Rapamycin Complex 1
NUFIP1: Nuclear FMR1 Interacting Protein 1
PD: Parkinson's Disease
PE: Phosphatidylethanolamine
PI3K: Phosphoinositide 3-kinase
PI3P: Phosphatidylinositol 3-phosphate
PTEN: Phosphatase and Tensin Homolog
ROS: Reactive Oxygen Species
SNARE: Soluble N-ethylmaleimide-sensitive-factor attachment protein receptor
TGN: Trans-Golgi Network
TNF: Tumour Necrosis Factor
TRAIL: Tumor Necrosis Factor-related Apoptosis-inducing Ligand
ULK1: Unc-51-like Autophagy Activating Kinase 1
WIPIs: WD-repeat Domain Phosphoinositide-interacting Proteins
ZF: Zinc Finger
